# Malaria Diagnosis and Hospitalization Trends, Brazil

**DOI:** 10.3201/eid1310.070052

**Published:** 2007-10

**Authors:** Patricia D. Santos-Ciminera, Donald R. Roberts, Maria das Gracas C. Alecrim, Monica R.F. Costa, Gerald V. Quinnan

**Affiliations:** * Uniformed Services University of the Health Sciences, Bethesda, Maryland, USA; † Fundacao de Medicina Tropical do Amazonas, Manaus, Brazil

**Keywords:** Malaria, epidemiology, Manaus, Amazonas, Brazil, Plasmodium vivax, severe disease, dispatch

## Abstract

We focused on rates of malaria in the state of Amazonas and city of Manaus, Brazil. *Plasmodium vivax* accounted for an increased number and rate of hospital admissions, while *P. falciparum* cases decreased*.* Our observations on malaria epidemiology suggest that the increased hospitalization rate could be due to increased severity of *P. vivax* infections.

## Abstract 2

Malaria Diagnosis and Hospitalization Trends, Brazil

The study of malaria prevalence in the state of Amazonas and city of Manaus indicates an increase in the percentage of hospitalized *Plasmodium vivax* patients and an overall increase in malaria cases caused by this parasite. Our observations on malaria epidemiology and case treatment suggest that the increased hospital admissions are associated with a higher frequency of severe disease associated with *P. vivax* infections. Amazonas includes most of the Brazilian Amazon Region, where malaria has been controlled but never eradicated. Since the 1980s, there has been a reemergence of malaria, which appears to coincide with changing malaria control policies associated with the ending of the Malaria Eradication Campaign (*1**,**2*).

From January through August 2003, the number of cases nationwide was reduced by 2.6%, when compared with the same period in 2002. However, this change did not represent a uniform reduction in the number of malaria infections within the country. The states of Amazonas, Rondônia, and Tocantins reported increases of 82.9%, 14.7%, and 10.3%, respectively (*3*). Perhaps the best indicator of what has been occurring with malaria control during the past 5 years is reflected in recent statistics for malaria in Amazonas and the city of Manaus. During the years of 2002 and 2003, the number of malaria cases reported in Amazonas increased 103.3% (*4*).

An observational study conducted in the reference center for diagnosis and treatment of malaria in Amazonas (Fundação de Medicina Tropical do Amazonas [FMT-AM]) described severe disease, including thrombocytopenia with hemorrhagic manifestations during infection with *P. vivax*. In that series, 46 (61.3%) of 75 patients admitted to the hospital for treatment of *P. vivax* malaria were classified with severe disease using predetermined criteria (*5*). We considered increased case severity as the need to hospitalize patients for treatment. Our primary goals were to present the epidemiology of malaria in Amazonas and the city of Manaus from 1980 to 2006 and to describe the overall rates, prevalence, and admission rates of malaria caused by *P. falciparum* and *P. vivax*.

## The Study

We extracted total yearly cases of malaria and population size in Amazonas from the database maintained by the Brazilian Ministry of Health (DATASUS, 2004), National Foundation of Health (*6**–**8*), and Secretary of Surveillance in Health (*3**,**9*). Data from FMT-AM were extracted from the malaria logbooks (for the years before the Foundation started publishing the reports) and from the Quarterly reports (for the years that the Foundation published the reports). All malaria cases diagnosed and referred for treatment are maintained (1989–1994) and quarterly reports are published by the FMT-AM (*10*). Quarterly reports published from 1995 to 2004 provided the total number of malaria diagnoses, case-patients admitted to the hospital, and number of deaths. Data from 2005 and 2006 were obtained by one of the authors (M.R.F. Costa) directly at FMT-AM (Subgerência de Arquivos Médicos e Contas Hospitalares).The hospital protocol is to exclude mixed infections by additional testing. We collected and tabulated data from these sources by year, parasite species, admissions, and percent admissions (Table). Percent admission was calculated as the total number of case-patients admitted to the hospital due to the specified parasite, divided by the total number of malaria cases caused by that same parasite in FMT-AM during that year, multiplied by 100.

**Table:** Total malaria cases in the state of Amazonas, Brazil, 1980–2006, and malaria case-patients diagnosed and admitted at FMT-AM by parasite, 1989–2006 *

Year	Amazonas†	FMT–AM							
		Malaria, all causes	*Plasmodium falciparum*			*P. vivax*			Other causes‡
			No. case-patients	No. admitted (%)		No. case-patients	No. admitted (%)		No. case-patients
1980	4,447	–	–	–		–	–		–
1981	8,169	–	–	–		–	–		–
1982	13,142	–	–	–		–	–		–
1983	10,299	–	–	–		–	–		–
1984	8,528	–	–	–		–	–		–
1985	11,196	–	–	–		–	–		–
1986	15,319	–	–	–		–	–		–
1987	15,233	–	–	–		–	–		–
1988	19,392	–	–	–		–	–		–
1989	34,944	4,347	1,262	264 (20.92)		3,043	26 (0.85)		42
1990	28,479	3,037	839	175 (20.86)		2,175	15 (0.69)		23
1991	45,849	5,765	664	179 (26.96)		5,076	23 (0.45)		25
1992	37,885	5,083	670	118 (17.61)		4,398	29 (0.66)		15
1993	55,364	10,157	2,834	325 (11.47)		7,284	24 (0.33)		39
1994	68,287	7,469	1,433	199 (13.89)		5,948	44 (0.74)		88
1995	52,602	5,765	1,049	174 (16.59)		4,518	30 (0.66)		198
1996	70,044	6,206	1,333	201 (15.08)		4,686	18 (0.38)		187
1997	94,382	10,483	1,871	186 (9.78)		8,506	175 (2.06)		106
1998	114,748	10,854	1,751	217 (12.39)		9,004	116 (1.29)		99
1999	167,722	19,967	4,459	341 (7.65)		15,238	155 (1.02)		270
2000	96,026	12,266	2,541	177 (6.97)		9,227	147 (1.59)		498
2001	48,385	4,315	813	127 (15.62)		3,443	95 (2.76)		59
2002	70,223	88,711	992	106 (10.69)		7,808	263 (3.37)		71
2003	143,343	30,017	2,213	150 (6.78)		27,679	677 (2.45)		125
2004	152,440	27,169	5,727	257 (4.49)		21,228	345 (1.63)		214
2005	229,330	31,243	8,698	264 (3.52)		22,174	378 (1.70)		371
2006	190,378	16,182	3,363	175 (4.31)		12,672	161 (1.27)		147

*2005–2006 data obtained at the Malaria Laboratory and Epidemiology Department of the FMT–AM by M.R.F.C. FMT-AM, Fundação de Medicina Tropical do Amazonas; –, data not available. †Total malaria cases in the state of Amazonas. ‡Includes *P. malariae* infections and mixed infections (*P. falciparum* + *P. vivax*).

**Figure 1:**
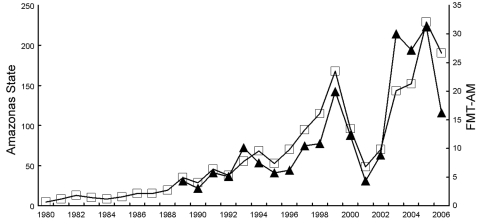
Malaria diagnoses (in thousands) according to blood smears positive for *Plasmodium falciparum* or *P. vivax* in Amazonas, Brazil (open squares), and Fundação de Medicina Tropical do Amazonas (FMT-AM) (solid triangles), 1980–2006.

Malaria cases from all causes in Amazonas, 1980–2006, are presented in Figure 1. An irregular increase is noted since 1988, reaching a peak in 1999, followed by a decline in 2001, only to rise again in the following years. A decrease was observed in 2006, but the data are not final. Figure 1 also shows the total number of malaria cases diagnosed at FMT-AM; fluctuations observed are very similar in direction and relative magnitude to those found statewide.

**Figure 2:**
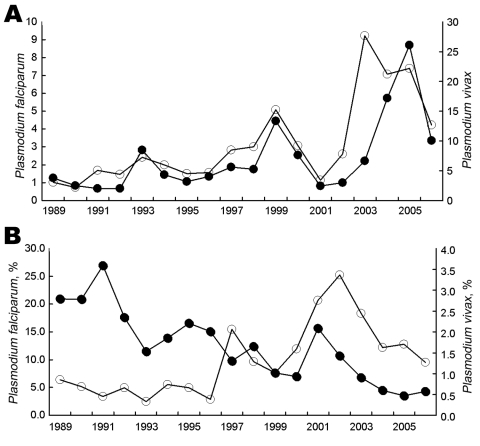
Malaria diagnoses by parasite species, *Plasmodium falciparum* (solid circles) and *P. vivax* (open circles), at the Fundação de Medicina Tropical do Amazonas (FMT-AM), Amazonas, Brazil, 1989–2006. A) Total numbers of diagnoses (in thousands). B) Percentage of infections resulting in hospital admission.

The number of infections due to *P. falciparum* and *P. vivax* diagnosed annually at FMT-AM are shown in Figure 2, panel A. The percentage of diagnosed case-patients admitted to the hospital, by parasite and year, is presented in Figure 2, panel B. In 1989, 264 (20%) of the patients with a diagnosis of *P. falciparum* infection were admitted to the hospital, while only 26 (0.85%) of those infected with *P. vivax* required admission. While *P. falciparum* remains the main cause of malaria admissions, we observed a significant increase in *P. vivax* admissions: the mean percent admissions from 1989 to 1996 was 0.59% (standard deviation [SD] 0.18), increasing to 1.91% (SD 0.74) from 1997 to 2006. This relative increase in *P. vivax* malaria requiring admission to the hospital for treatment was disproportionate to the change in numbers of cases and to the relative frequency of *P.* *vivax* cases over *P.* *falciparum* malaria cases.

## Conclusions

We presented the epidemiology of recent malaria cases in the State of Amazonas and city of Manaus, emphasizing the emergence of severe *P.* *vivax* malaria. Assuming that patients requiring hospital admission were sicker than those treated as outpatients, we observed that malaria transmission in this region was continuous and fluctuated in intensity. *P. vivax* was consistently the main cause of malaria, but the number of patients with *P. vivax* requiring hospital admission increased significantly in recent years. Changes in control operations were linked to the reestablishment of malaria in major urban areas of the Amazon basin, e.g., Belém (*11*). In Manaus, this could have had an impact on *P. vivax* disease manifestations and severity but did not seem to have affected the severity of disease caused by *P. falciparum,* perhaps because the current policy of early diagnosis and treatment has been reported to have a greater impact on disease caused by *P. falciparum* than *P. vivax* (*12**,**13*).

In this study, we assumed that case definition and criteria for admission at FMT-AM, for both *P. vivax* and *P. falciparum* malaria, were relatively constant (http://www.fmt.am.gov.br/). Our data showed that the likelihood of hospital admissions for case-patients diagnosed with *P. vivax* malaria increased substantially after 1996, while the percentage of *P. falciparum* admissions declined. The decreasing rate of admission for *P. falciparum* malaria during the later years of our study supports the interpretation that the criteria for admission to FMT-AM were not loosened. It is possible that referrals to FMT-AM from elsewhere in the region increased during this period, but that would likely affect *P. falciparum* admissions too. Based on these considerations, we interpret the data as suggestive of an increased illness associated with *P. vivax* infections in the region.

In this study we did not attempt to describe the specific disease manifestations that were the basis for admissions of individual patients. However, recent reports described a range of unusual manifestations of *P. vivax* infection elsewhere (*14*), consistent with the disease manifestations reported in Manaus (*5*).

Biologic aspects of the human host, vector, and parasite and changes in the environment contribute to the epidemiology of malaria. Our data demonstrate that malaria is a growing health burden in the Amazon Region of Brazil and that disease caused by *P. vivax* is a substantial and increasing threat to the health of the population in Manaus. More studies are needed to understand the complex mechanisms of this disease and its impact on susceptible populations.

## References

[1] Brazil: Ministério da Saúde, Fundação Nacional de Saúde (FUNASA). Vigilância Epidemiológica: Programa Nacional de Prevenção e Controle da Malária–PNCM. Brasília-DF. Dec 2002. [cited 2007 Aug 29]. Available from http://www.funasa.gov.br

[2] Loiola CCP, da Silva CJM, Tauil PL. Controle da malaria no Brasil: 1965–2001. Rev Panam Salud Publica. 2002;11:235–44. 10.1590/S1020-49892002000400005 12049032

[3] Brasil, Ministério da Saúde, Secretaria de Vigilância em Saúde (SVS). Boletim Epidemiológico da Malária. No. 2. Dec 2003. [cited 2007 Aug 29]. Available from http://dtr2001.saude.gov.br/svs/epi/malaria/pdfs/be_malaria_02_2003.pdf

[4] Fundação de Medicina Tropical do Amazonas (FMT–AM), 2005. Informe Epidemiológico, No. 1. 2005. Malária notificada no Amazonas no Período de 2003 a 2004.

[5] Alecrim MGC. Estudo Clínico, resistência e polimorfismo parasitário na malária pelo *Plasmodium vivax*, em Manaus. Brasília-DF: 2000. Universidade de Brasília; Faculdade de Medicina/Núcleo de Medicina Tropical, Tese de Doutorado. p. 177.

[6] Brazil: Fundação Nacional de Saúde (FUNASA). Vigilância Epidemiológica: Situação da Prevenção e Controle das Doenças Transmissíveis no Brasil. Brasília-DF. Sep 2002:22–3. [cited 2007 Aug 29]. Available from http://www.funasa.gov.br

[7] Brazil: Fundação Nacional de Saúde (FUNASA). Vigilância Epidemiológica. Brasília-DF. 2004. [cited 2007 Aug 29]. Available from http://www.funasa.gov.br

[8] Brasil: Fundação Nacional de Saúde (FUNASA). Vigilância Epidemiológica: Casos confirmados, segundo o período de diagnóstico e local de residência, por U.F. Brasil, 1980–2001. Brazil-DF. 2003. [cited 2007 Aug 29]. Available from http://www.funasa.gov.br

[9] Brasil: Secretaria de Vigilância em Saúde (SVS). Série histórica de casos de Doenças de Notificação compulsória-Amazonas, 1980–2001. Brasília-DF. 2004. [cited 2007 Aug 29]. Available from http://dtr2001.saude.gov.br/svs/epi/situacao doencas/transmissiveis00.htm

[10] Fundação de Medicina Tropical do Amazonas (FMT–AM). Boletim trimestral, Números I a X. Manaus, Amazonas, Brasil: 1995 to 2004.

[11] Libonati RM, Dos Santos MVN, Pinto AYN, Calvosa AM, Ventura PHM, Figueiredo JM, Malária autoctone na Grande Belém: panorama atual e prevalência no últimos seis anos. Rev Soc Bras Med Trop. 2000;33(Suppl 1):347.10936947

[12] Mendis K, Sina BJ, Marchesini P, Carter R. The neglected burden of *Plasmodium vivax* malaria. Am J Trop Med Hyg. 2001;64:97–106. 11425182 10.4269/ajtmh.2001.64.97

[13] Pan American Health Organization. Situation of malaria programs in the Americas. Epidemiol Bull/PAHO. 2001;22:10–4. 11370647

[14] Kochar DK, Saxena V, Singh N, Kochar SK, Kumar SV, Das A. *Plasmodium vivax* malaria. Emerg Infect Dis. 2005;11:132–4. 15705338 10.3201/eid1101.040519 PMC3294370

